# Advertisement and knowledge of tobacco products among Ellisras rural children aged 11 to 18 years: Ellisras Longitudinal study

**DOI:** 10.1186/1471-2431-13-111

**Published:** 2013-08-02

**Authors:** Kotsedi D Monyeki, Han CG Kemper, Lateef O Amusa, Marcus Motshwane

**Affiliations:** 1 Department of Physiology, University of Limpopo, P.O. Box 130, Pretoria 0204, South Africa; 2 Medical University of South Africa Campus, P.O. Box 130, Pretoria 0204, South Africa; 3 VU University Medical Centre, The Institute for Health and Care Research (EMGO+), Amsterdam, the Netherlands; 4 Centre for Biokinetics, Recreation and Sport Science, University of Venda, Private Bag x 5050, Thohoyandou 0950, South Africa; 5 Department of statistics, University of Limpopo, P.O. Box 130, Pretoria 0204, South Africa

**Keywords:** Advertisement, Tobacco use, Youth, Knowledge of tobacco, Comprehension

## Abstract

**Background:**

Tobacco products use is the leading cause of chronic diseases morbidity and mortality. This study explores an exposure to tobacco advertisements factors and knowledge, an association with snuff/pipe usage and cigarette smoking among Ellisras rural children aged between 11 to 18 years.

**Methods:**

A total of 1654 subjects (854 boys and 800 girls) who were part of the Ellisras Longitudinal Study completed the questionnaire.

**Results:**

A significant (p < 0.05) number of boys (11.7%) compared to girls (8.8%) received free cigarettes from the members of the community. Bill boards were successful in advertising tobacco products among the Ellisras rural boys (17% boys and 12.8% for girls, p < 0.022). Multivariate analyses found significant association between cigarette smoking (OR = 1.7 95%CI 1.1-2.7 and Model 2 OR 1.6 95%CI 1.0-2.6 adjusted for age and gender) and advertisements of tobacco products on the TV screens, videos or movies.

**Conclusions:**

Exposure to tobacco products advertisements was high among Ellisras rural children. Though tobacco products legislation exists in South Africa, efforts should be taken by the health professionals to emphasize the danger of using tobacco products even among the illiterate. Teachers and parents should refrain from advertising tobacco products at schools and at homes.

## Background

The use of tobacco products is addictive although it is a preventable risk factor for people with chronic diseases [1,2]. The latency from the onset of the first cigarette puff to the nicotine dependence was lower among adolescents compared to adults [3-5]. It is disturbing to note that despite intensifying regulations [6,7], children and adolescents continue to be exposed to tobacco products advertisement in magazines [8-11], at the point of sale [12] and in interactive media like video movies [8,13,14]. However, although we can conclude that exposure to tobacco products advertisements leaves a complex and positive traces in the minds of children and adolescents, it remains unclear whether these traces provide insight knowledge to the danger of tobacco products usage, or whether they may relate to tobacco products usage later in life.

Pictures of tobacco products, brand names and logos are recognized and understood by children under three years of age as advertised [15,16]. For example, Benson & Hedges advertisements create the perception that people who smoke the brand are relaxed, interesting, cool and rich [16]. In the present study sample, the prevalence of tobacco products usage among Ellisras rural boys starts at an early age and increases (4.9 to 17.1%) with age (11–12 years to 17 to 18 years) while no Ellisras rural girls of the same age group smoke cigarette but use snuff (smokeless tobacco) (0.7 to 4.1%) [17]. In considering the escalating tobacco products usage among the youth, the effect of tobacco advertisements and the knowledge of use on different products brand will shed more light on the future risks of tobacco products addiction. The purpose of this study was therefore to investigate cross-sectionally the 1) Effects of tobacco products advertisements and 2) knowledge of tobacco products among Ellisras rural children aged between 11 to 18 years who are part of the Ellisras Longitudinal Study (ELS). Additionally, 3) the risks of future use of tobacco products was investigated.

## Methods

The geographical area, research design and sampling for the ELS was explained in detail elsewhere [17]. Briefly, a sample of the current study consisted of 1654 children (854 boys and 800 girls) aged between 11 to 18 years who are part of the ELS. The study was carried out during the period 1 March to 3 May 2005 from the 1771 (923 boys and 848 girls) who participated in the anthropometric measurements of November 2003. The Ethics Committee of the South African Medical Research Council granted ethical approval prior to the survey and the parents or guardians provided informed consent. The children signed the assent form after receiving verbal assent from the project principal investigator (KDM).

### Data collection

Questions addressing relevant concepts (knowledge, practice, attitudes, beliefs and advertisements) of the present study were extracted from the previously validated questionnaires [1,2,18,19]. An expert panel was then convened to recommend which questions should be included in the advertisement and knowledge section. The principal investigator with the help of the Ellisras local teachers translated the questionnaire from English into the two locally spoken languages (Northern Sotho and Tswana) and then translated it back to English. The back translation to English showed no disparity with the Northern Sotho and Tswana languages.

### Definition of concepts

A smoker was defined as anyone who smoked at least one cigarette or any other type of tobacco products like pipe, snuff, home-made tobacco or indigenous tobacco per day at the time of the survey. Snuff is a powder-like tobacco product stored in a 15 g container called “Thekgwane”. Those who never used tobacco products at the time of the survey were considered non smokers. Advertisement of tobacco products was defined as any utilitarian objects bearing a tobacco products logo or brand on or the visibility of any form of tobacco products logo or brand name. In the interview, participants were asked how strongly they believed on certain statements regarding tobacco knowledge.

### Quality control

All selected field workers underwent intensive training for one week prior to the survey. The inter-tester (between fieldworkers) and intra-tester (principal investigator and field workers) technical error of measurements ranged from 97 to 100% in agreement with the coding of the advertisement responses of the smoking questionnaires. Questionnaires relating to the knowledge of tobacco, however, range from 95 to 98% agreement.

### Data analysis

All analyses were performed using the SPSS Version 14.0 (SPSS Inc., Chicago, IL, USA). Analyses were run for descriptive statistics by gender and age. Chi-square test was used to compare sets of nominal data that had larger frequency counts whereas the Fisher’s exact test was used when the frequency counts were small (less than five) between genders. Binary logistic regression analysis was used to study the factors associated with tobacco products usage. Firstly, univariate analysis (model 1) was undertaken for each covariate (knowledge and advertisement variables) with cigarette smoking and snuff/pipe usage as dependent variables. Secondly, in multivariate analysis (Model 2) the significant association at the univariate level was studied for covariate (knowledge and advertisement variables) adjusted for age and gender with cigarette smoking and snuff/pipe use as dependent variables. Only significant association expressed as odds ratios and the 95% confidence interval were reported. The statistical significance was set at p < 0.05.

## Results

Table 1 presents descriptive statistics for the advertisement of tobacco products as reported by Ellisras rural children aged between 11 to 18 years. A significant (p < 0.054) number of boys (11.7%) compared to girls (8.8%) has received free cigarettes gifts from the Ellisras rural community members. A number of boys (26.3%) and girls (21.9%) reported seeing tobacco products being advertised in their school classrooms. A significant (P ranging from 0.008 to 0.022) number of Ellisras boys reported to have seen few advertisements of tobacco products at home (21.0% boys and 17.5% girls) and on the bill boards (boys = 17.0% and girls = 12.8%).

**Table 1:** **Descriptive statistics for advertisement of tobacco product as reported by Ellisras rural children** (**boys** = **854 and girls** = **800**) **aged 11 to 18 years**

**Statement**	**Boys**	**Girls**	**P**-**value**
	**(n)%**	**(n) %**	
1. When you watch TV, Video or movies, how often do you see actors smoking or chewing tobacco? A I never watch TV, video, movie	(420) 49.2	(393) 49.1	0.512
A lot	(229) 26.8	(210) 26.3	0.443
Sometimes	(205) 24.0	(197) 24.6	0.431
2. Have you ever been given free cigarettes by anybody other than your parents, friends or relatives? Yes	(100) 11.7	(70) 8.8	0.044
No	(754) 88.3	(730) 91.3	0.335
3. Do you have something (t-shirt, pen, backpack) with a cigarette brand or any tobacco brand logo on it? Yes	(35) 4.1	(45) 5.6	0.103
No	(819) 95.9	(755) 94.4	0.431
4. During the past 30 days how many advertisements for cigarettes or other tobacco products have you seen on the billboards? None	(488) 57.1	(489) 61.1	0.214
A lot	(145) 17.0	(102) 12.8	0.022
Few	(221) 25.9	(209) 26.1	0.487
5. During the past 30 days how many advertisements or promotions for cigarettes or other tobacco products have you seen in newspapers or magazines? A lot	(135) 15.8	(126) 15.8	0.516
A few	(315) 36.9	(279) 34.9	0.296
None	(404) 47.3	(395) 49.4	0.325
6. During the past 30 days how many advertisements or promotions for cigarettes or other tobacco products have you seen in a classroom? A lot	(84) 9.8	(62) 7.8	0.100
A few	(141) 16.5	(113) 14.1	0.139
None	(629) 73.7	(625) 78.1	0.227
7. During the past 30 days how many advertisements or promotions for cigarettes or other tobacco products have you seen at home? A lot	(94) 11.0	(88) 11.0	0.530
A few	(179) 21.0	(140) 17.5	0.008
None	(581) 68.0	(572) 71.5	0.271

Table 2 presents descriptive statistics for knowledge about tobacco products among Ellisras rural children aged between 11 and 18 years. Ellisras rural children (80.3% for boys and 75.4% for girls) knew that smoking was harmful to ones health at an early ages (11–12 years). There was a significant difference (p < 0.05) between boys (14.6%) and girls (8.2%) in the age groups 13–14 years on the fact that passive smoking had a negative effect on one’s health. A significant (p < 0.05) higher number of girls (32.1%) compared to boys (19.0%) in the age groups 11–12 years believed that it does not help giving up smoking when you are old since the damage has already been done.

**Table 2:** Descriptive statistics for knowledge about tobacco products among Ellisras rural children aged 11 to 18 years

**Number**	**Statement**	**Age range**							
		11-12 years		13-14 years		15-16 years		17-18 years	
		Boys	Girls	Boys	Girls	Boys	Girls	Boys	Girls
		N = 142	N = 134	N = 205	N = 196	N = 308	N = 294	N = 199	N = 176
		% (n)	% (n)	% (n)	% (n)	% (n)	% (n)	% (n)	% (n)
1a	Smoking is harmful to one’s health	80.3(114)	75.4(101)	73.7(151)	77.0(151)	79.9(246)	79.9(235)	74.9(149)	77.3(136)
1b	Smoking is good for your health	7.7(11)	10.4(14)	10.7(22)	6.6(13)	5.8(18)	7.1(21)	7.0(14)	9.1(16)
1c	Smoking has no effect on one’s health	12.0(17)	14.2(19)	15.6(32)	16.3(32)	14.3(44)	12.9(38)	18.1(36)	13.6(24)
2a	Smoking is bad for women who are pregnant	62.7(89)	64.9(87)	62.4(128)	62.8(123)	67.9(209)	72.1(212)	71.4(142)	69.3(122)
2b	Smoking is good for women who are pregnant	14.8(21)	16.4(22)	11.7(24)	15.3(30)	12.0(37)	9.9(29)	8.5(17)	11.4(20)
2c	Smoking has no effect on women who are pregnant	22.5(32)	18.7(25)	25.9(53)	21.9(43)	20.1(62)	18.0(53)	20.1(40)	19.3(34)
3a	Smoking near you is harmful to your health	72.5(103)	70.1(94)	74.6(153)	84.2(165)	83.1(256)	83.7(246)	86.4(172)	80.1(141)
3b	Smoking near you is good for your health	12.7(18)	12.7(17)	10.7(22)	7.7(15)	7.1(22)	6.8(20)	7.0(14)	6.8(12)
3c	Smoking has no effect on your health	14.8(21)	17.2(23)	14.6(30)*	8.2(16)*	9.7(30)	9.5(28)	6.5(13)*	13.1(23)*
4a	Chewing tobacco is much healthier than smoking cigarettes	23.2(33)	17.9(24)	23.4(48)	16.8(33)	19.5(60)	16.7(49)	17.1(34)	14.2(25)
4b	Chewing tobacco is not healthier than smoking cigarettes	43.0(61)	49.3(66)	34.6(71)*	48.5(95)*	40.3(124)	38.1(112)	37.2(74)	40.9(72)
4c	There is no difference between chewing tobacco smoking cigarettes they are all bad	33.8(48)	32.8(44)	42.0(86)	34.7(68)	40.3(124)	45.2(133)	45.7(91)	44.9(79)
5a	There is no harm to your health in taking snuff.	28.9(41)	26.1(35)	21.5(44)	18.9(37)	25.3(78)	23.8(70)	22.1(44)	25.6(45)
5b	It is bad for your health to take snuff	58.5(83)	60.4(81)	62.9(129)	70.9(139)	65.3(201)	65.0(191)	66.8(133)	62.5(110)
5c	It has no effect on your health	12.7(18)	13.4(18)	15.6(32)	10.2(20)	9.4(29)	11.2(33)	11.1(22)	11.9(21)
6a	Snuff is good for women who are stressed.	19.0(27)	25.4(34)	19.0(39)	15.3(30)	23.1(71)	19.0(56)	17.6(35)	15.9(28)
6b	Snuff is not good for women who are stressed	61.3(87)	44.0(59)	46.8(96)	48.5(95)	50.3(155)	50.3(148)	47.7(95)	45.5(80)
6c	Snuff has no effect on women who are stressed	19.7(28)	30.6(41)	34.1(70)	36.2(71)	26.6(82)	30.6(90)	34.7(69)	38.6(68)
7a	Smoking a pipe is healthier than smoking cigarettes	10.6(15)	11.9(16)	9.8(20)	10.2(20)	9.1(28)	10.2(30)	7.5(15)	9.1(16)
7b	Smoking a pipe is not healthier than smoking cigarettes	65.5(93)	67.2(90)	68.8(141)	64.8(127)	65.3(201)	65.0(191)	61.3(122)	56.8(100)
7c	There is no difference smoking a pipe or cigarettes they are all bad	23.9(34)	20.9(28)	21.5(44)	25.0(49)	25.6(79)	24.8(73)	31.2(62)	34.1(60)
8a	It doesn’t help giving up smoking when you are old, since the damage has been done	19.0(27)*	32.1(43)*	27.8(57)	24.5(48)	20.1(62)	22.4(66)	23.1(46)	21.6(38)
8b	It does help giving up smoking when you are old, since the damage can be stopped	40.1(57)	27.6(37)	28.8(59)	31.6(62)	38.6(119)	33.0(97)	32.2(64)	35.8(63)
8c	It doesn’t matter whether you stop or don’t stop smoking	40.8(58)	40.3(54)	43.4(89)	43.9(86)	41.2(127)	44.6(131)	44.7(89)	42.6(75)
9a	Smoking cigarettes is only bad when you smoke more than 20 a day	53.5(76)	40.3(54)	31.2(64)	39.3(77)	32.1(99)	35.4(104)	28.6(57)	26.7(47)
9b	Smoking less than 20 cigarettes a day is also bad for you	29.6(42)	34.3(46)	41.5(85)	38.8(76)	37.7(116)	34.4(101)	40.7(81)	41.5(73)
9c	It doesn’t matter how many you smoke or don’t smoke	16.9(24)	25.4(34)	27.3(56)	21.9(43)	30.2(93)	30.3(89)	30.7(61)	31.8(56)

*P < 0.05.

Table 3 presents a significant odds ratio and 95% confidence interval for the association of cigarette smoking, snuff/pipe use, advertisement and knowledge of tobacco products among Ellisras rural children. Tobacco products usage, either by cigarette smoking (OR 1.6 95%CI 1.1-2.6 and OR 0.7 95%CI 0.4 to 1.1 adjusted for age and gender) or snuff/pipe usage (Model 1 = OR 0.6 95% CI 0.3- 0.9 and Model 2 = OR 0.5 95% CI 0.3-0.9 adjusted for age and gender) were reported to have negative effects on pregnant women. The Ellisras children also reported the danger of being next to the person who use tobacco products; that is, either cigarette smoking (Model 1 = OR 0.6 95%CI 0.4-1.0 and Model 2 OR 2.1 95%CI 1.1-4.0 adjusted for age and gender) or snuff/pipe usage (Model 1 = OR 2.3 95%CI 1.2 4.4, and Model 2 = OR 2.3 95%CI 1.1- 4.5 adjusted for age and gender). There was a significant association between cigarette smoking (Model 1 OR = 1.7 95%CI 1.1-2.7 and Model 2 OR 1.6 95%CI 1.0-2.6 adjusted for age and gender) and advertisements of tobacco products on the TV screens, videos or movies. Snuff/pipe usage (Model 2 OR = 2.5 95%CI 1.4 to 4.5 adjusted for age and gender) was significantly associated with viewing tobacco products advertisement on the bill boards.

**Table 3:** **Odds ratio and 95**% **confidence interval for cigarette smoking**, **snuff** /**pipe use by knowledge of tobacco products and advertisement of tobacco products for Ellisras rural children aged 11 to 18 years**

	**Cigarette smoking**		**Pipe and snuff**	
	**Model 1**	**Model 2**	**Model 1**	**Model 2**
	**OR(95%CI)**	**OR(95%CI)**	**OR(95%CI)**	**OR(95%CI)**
**Knowledge of tobacco products**				
Smoking has no effect on one’s health	1.9(1.1-3.1)	1.9(1.1-3.2)	-	-
Smoking is bad for women who are pregnant	1.6(1.0-2.6)	0.7(0.4-1.1)	0.4(0.3-0.7)	0.4(0.3-0.7)
Smoking near you is harmful to your health	()	0.5(0.3-0.9)	0.6(0.3-0.9)	0.5(0.3-0.9)
Chewing tobacco is much healthier than smoking cigarettes	-	-	4.5(2.7-7.4)	4.5(2.7-7.4)
There is no difference between chewing tobacco smoking cigarettes they are all bad	-	-	-	0.5(0.3-0.9)
Snuff is good for women who are stressed.	-	-	-	0.5(0.3-0.8)
It doesn’t help giving up smoking when you are old, since the damage has been done	-	-	-	1.7(1.0-2.9)
**Advertisement of tobacco products**				
1. When you watch TV, Video or movies, how often do you see actors smoking or chewing tobacco? A lot	1.7(1.1-2.7)	1.6(1.0-2.6)	-	-
2. Have you ever been given free cigarettes by anybody other than your parents, friends or relatives? Yes	-	4.0(2.4-6.7)	-	3.8(2.2-6.7)
3. Do you have something (t-shirt, pen, backpack) with a cigarette brand or any tobacco brand logo on it? Yes			-	2.5(1.1-5.7)
4. During the past 30 days how many advertisement for cigarette or other tobacco products have you seen on the billboards? A lot		3.4(2.1-5.6)	-	2.5(1.4-4.5)
5. During the past 30 days how many advertisements or promotions for cigarettes or other tobacco products have you seen in a classroom? A lot		2.0(1.1-3.6)	-	2.4(1.2-4.5)
6. During the past 30 days how many advertisements or promotions for cigarettes or other tobacco products have you seen at home? None	0.3(0.2-0.5)	0.4(0.2-0.6)		-

Model 1 = crude, model 2 = adjusted for age and gender.

## Discussion

In this study, cross-sectional results of exposure to tobacco products advertisements and knowledge of tobacco products among Ellisras rural children aged 11 to 18 years who were part of the Ellisras Longitudinal Study were investigated. The results indicate high exposure to various forms of tobacco products advertising. The following factors increased the probability of tobacco products usage among adolescents: seeing actors on TV, movies using tobacco products, having something with tobacco products, seeing tobacco products on the bill boards, classrooms and at their homes. Similar factors were reported by Daku et al. [20].

Tobacco control legislation was introduced in South Africa more than a decade before this study was carried out. All tobacco products have health warnings. Rothmans was the most purchased brand after home- made tobacco in the current study [17]. It is worth to mention that in South Africa, Rothmans brand was the main elite popular (among black community) sport (soccer) sponsor. This was before tobacco restrictions advertisements came into force more than a decade, before the study was carried out. However, local business outlets still maintain the same advertisement in their bill boards which the youth interact with on a day to day basis at the point of sale. It was not surprising that the prevalence of tobacco products usage increases with age increasing in the current sample [17]. Furthermore, the prevalence of tobacco products usage among the youth was not only increasing in South Africa but also to some African states like Zimabwe, Ghana, Zambia and Nigeria to name but a few [18,20-23]. The possible explanation could be that brand user imagery could be the major positioning strategy that advertisers used to create positive attitude to their brands, hence increasing the likelihood of purchase [7,24,25].

Health warning dangers of using tobacco products are well conveyed on each tobacco product in South Africa. However, in this study the probability of tobacco products use and knowledge of the danger posed by tobacco products were evident from the following factors: smoking has no effect on one’s health, it is bad for pregnant women, being close to somebody who smokes is not healthy and that snuff is good for women who are stressed. Furthermore, this study discovered that social sources (friends, homes, classrooms and magazines) were the most common sources of acquisitions of tobacco products among Ellisras rural children. This was despite the fact that tobacco products had limited promotional avenues in South Africa [18,26] and tobacco products companies claimed that advertisements were not directed to children [24]. Similar results were reported by Unger et al. [27] among USA grade eight children and Donovan et al. [16] for Australian children aged 10 to 12 years.

The mechanism underlying tobacco products usage at both individual and population level is complex. Tobacco products use is influenced by the interaction of environment and social factors as well as an individual knowledge of tobacco products usage. Although there is tobacco products control policy in South Africa, illiteracy is a major challenge among rural population [28,29]. The findings of this study suggest that public awareness should be the first intervention by health professionals in terms of providing primary health care to these sectors of the community in an effort to reduce the initiation of tobacco products by the youth. Finally, the role of schools, in the teaching of health education and curbing the use of home- made tobacco products in the rural South African schools could be helpful.

This study involves children from rural areas of South Africa which narrows the demographics and the ongoing changing patterns of the South African population today. Furthermore, we did not consider the socio-economic status of families of the participants in the analysis. However, environmental factors such as excessive usage of alcohol and relaxed tobacco products regulation in rural areas [18,26,30-32] clearly affect youth’s exposure to tobacco products. The extent at which the youth understand the danger of tobacco products on their lifestyles were also not covered in the study. Lastly, although exposure to tobacco products seems unlikely to affect youth’s understanding and the ultimate use [23,24] certainly, the exact wording of the advertisement of the tobacco products seen by the Ellisras rural youth could influence future use of the products which we did not cover in our study. However, this study provided valuable information on tobacco products promotion and knowledge in rural South African children and could be followed by an intervention study to remove this particular public health problem from rural South African communities.

## Conclusions

Ellisras rural children were aware that tobacco products usage was harmful to their health. Binary logistic regression analysis revealed that having seen tobacco advertisements on TV and movies, billboards, home and school classroom, was positively associated with tobacco products use. Prevention strategies aimed at reducing tobacco usage among the youth should also be designed in such a way that they reduce the use of home made tobacco products. Parents and teachers must be advised to refrain from advertising tobacco products at their homes and in the school classrooms. Also, a follow-up on the use of tobacco products may shed more light on how smoking is related to other biological parameters in the Ellisras rural population.

## Competing interests

The authors declare that they have no competing interest.

## Authors’ contributions

KDM participated in the study design, literature search, drafting of the manuscript, critical revision of the manuscript for important intellectual content, administrative, technical, and material support, such as supervision of the study. HCGK participated in study design, critical revision of the manuscript for important intellectual content. LOA participated in critical revision of the manuscript for important intellectual content. MM participated in data analysis and critical evaluation of the manuscript. All the authors have read and approved the final version of the manuscript.

## Pre-publication history

The pre-publication history for this paper can be accessed here:

http://www.biomedcentral.com/1471-2431/13/111/prepub
